# *Lactobacillus fermentum* Postbiotic-induced Autophagy as Potential Approach for Treatment of Acetaminophen Hepatotoxicity

**DOI:** 10.3389/fmicb.2017.00594

**Published:** 2017-04-06

**Authors:** Miroslav Dinić, Jovanka Lukić, Jelena Djokić, Marina Milenković, Ivana Strahinić, Nataša Golić, Jelena Begović

**Affiliations:** ^1^Laboratory for Molecular Microbiology, Institute of Molecular Genetics and Genetic Engineering, University of BelgradeBelgrade, Serbia; ^2^Department of Microbiology and Immunology, Faculty of Pharmacy, University of BelgradeBelgrade, Serbia

**Keywords:** autophagy, *Lactobacillus fermentum*, postbiotics, acetaminophen, hepatotoxicity

## Abstract

The aim of this study was to investigate the potential of postbiotics originated from *Lactobacillus fermentum* BGHV110 strain (HV110) to counteract acetaminophen (APAP)-induced hepatotoxicity in HepG2 cells. This strain was selected according to its autophagy inducing potential, based on previous studies reporting protective role of autophagy in APAP caused cellular damage. Cell viability was assessed using MTT and LDH assays, while autophagy was monitored by qPCR analysis of *BECN1*, *Atg5*, *p62/SQSTM1*, and *PINK1* mRNA expression and by Western blot analysis of p62/SQSTM1 and lipidated LC3 accumulation. Our results showed that detrimental effect of APAP on cell viability was suppressed in the presence of HV110 which was linked with increased conversion of LC3 protein and p62/SQSTM1 protein degradation. Additionally, higher *p62/SQSTM1* and *PINK1* mRNA transcription were noticed in cells co-treated with APAP/HV110, simultaneously. In conclusion, this study suggests that HV110 enhances activation of PINK1-dependent autophagy in HepG2 cells and its eventual co-supplementation with APAP could be potentially used for alleviation of hepatotoxic side effects caused by APAP overdose.

## Introduction

Acetaminophen [paracetamol, *N*-acetyl-*p*-aminophenol (APAP)] is widely used analgesic and antipyretic drug which is safe and effective at a therapeutic dose (Lee, 2004). However, acute or cumulative overdose can cause hepatic necrosis and liver failure (Larson et al., 2005). The mechanisms of APAP-induced liver injury described to this moment include generation of reactive metabolite, *N*-acetyl-*p*-benzoquinone imine (NAPQI) and *p*-aminophenol (PAP) (Miyakawa et al., 2015). Given an important role of autophagy in elimination of damaged organelles, including mitochondria, it has been shown that activation of autophagy could serve as a cellular adaptive mechanism to counteract APAP-induced hepatotoxicity (Igusa et al., 2012; Ni et al., 2012).

Autophagy is tightly regulated and highly inducible catabolic cellular process involved in degradation of organelles and long-living proteins. During this process double-membrane vesicles (autophagosomes) are formed and fused with lysosomes while enclosed material is degraded. Literature data suggest that deregulation of autophagy is highly associated with various liver diseases. For example, in liver ischemia reperfusion injury autophagy exhibits prosurvival activity, while in hepatocellular carcinoma autophagy level is decreased (Rautou et al., 2010). Therefore, therapeutics capable to induce autophagy could be beneficial for liver associated pathological conditions.

In addition to well-known autophagy promoting stimuli (e.g., starvation, rapamycin, hormones), upregulation of autophagy can occur in bacterial, viral, and parasitic infections (Lum et al., 2005; Mizushima et al., 2010). Irving et al. (2014) demonstrated that peptidoglycan derived from *Helicobacter pylori* and *Pseudomonas aeruginosa* promotes autophagy in epithelial cells via NOD1 receptor activation. Moreover, several studies reported potential of some *Bifidobacteria* and *Lactobacillus* species to stimulate or suppress autophagy (Wu et al., 2013; Lin et al., 2014; Motevaseli et al., 2016).

Lactobacilli are beneficial bacteria, commonly used as probiotics. It has been shown that certain *Lactobacillus* strains were associated with suppression of liver injury caused by oxidative stress, pathogens, hepatic encephalopathy, and alcoholic liver disease (Segawa et al., 2008; Forsyth et al., 2009; Rishi et al., 2009). However, novel trends in probiotic supplementation are oriented toward replacement of live bacteria with non-viable bacterial extracts and metabolic by-products, termed postbiotics (Konstantinov et al., 2013; Patel and Denning, 2013). This new approach reduces health risks associated with consumption of live bacteria, especially concerning their high immune stimulating potential (Tsilingiri et al., 2012). Recent data showed that postbiotics can modulate different cellular pathways. Sharma et al. (2011) reported the cyto-protective activity of supernatants obtained from probiotics *Enterococcus lactis* IITRHR1 and *Lactobacillus acidophilus* MTCC447 against APAP induced hepatotoxicity. Particularly, the authors showed that the postbiotics have potential to restore glutathione level, reduce generation of major oxidative stress markers and to enhance production of anti-apoptotic (Bcl-2) protein.

Considering the fact that mitochondrial damage is a critical event in APAP-induced oxidative stress and cellular necrosis, activation of PINK1-Parkin signaling pathway is crucial for upregulation of mitochondrial autophagy. PINK1 is required for Parkin recruitment to damaged mitochondria when mitochondrial membrane potential is impaired, causing recruitment of p62/SQSTM1, an autophagy adaptor molecule, which is essential for final mitochondrial clearance (Williams and Ding, 2015).

In the light of above presented facts, we assessed the potential of autophagy inducing postbiotics originated from *Lactobacillus fermentum* BGHV110 strain to improve the viability of human hepatoma HepG2 cells exposed to APAP. Hence, according to our knowledge the results of this study for the first time suggest on the cyto-protective effect of postbiotics in APAP mediated hepatotoxicity.

## Materials and Methods

### Bacterial Strain and Preparation of the Bioactive Lysate (Postbiotic)

*Lactobacillus fermentum* BGHV110, a human isolate from Laboratory collection, was used in the study. Determination of the species identity was performed by 16S rDNA sequencing using UNI16SF and UNI16SR primers complementary to 16S rDNA (Jovcic et al., 2009). PCR amplification was performed using KAPA *Taq* DNA polymerase kit (Kapa Biosystems, Wilmington, MA, USA). Reaction mixture contained: 20 mM Tris-HCl (pH 8.4), 50 mM KCl, 3 mM MgCl2, 50 mM each of the dNTPs, 1 U of *Taq* polymerase, 5 pM of each primer (for multiplex PCR 0.25 μM of each primer), and 0.1 μg of template DNA in a final volume of 50 μl. The PCR product was purified with QIAquick PCR Purification KIT (Qiagen, Hilden, Germany) and sequenced by Macrogen (Seoul, South Korea). The BLAST algorithm^1^ was used to determine the most related DNA sequences in the NCBI GenBank database.

Bacteria were cultured in MRS broth (Merck, Darmstadt, Germany) at 37°C under anaerobic conditions using Anaerocult A (Merck). In order to obtain bioactive lysate overnight culture was pelleted (5000 rpm, 10 min) and washed twice with phosphate-buffered saline (PBS). Bacterial pellet was 10 times concentrated in PBS followed by homogenization in a French press (three passages). Homogenized bacterial suspension was lyophilized (Alpha 1–4 LSC Plus Freeze dryer, Martin Christ, Germany) and stored at +4°C until further use.

### Cell Culture and Treatments

Human hepatoma cell line HepG2 was cultured in low glucose DMEM supplemented with 10% fetal bovine serum (FBS), 100 U/ml penicillin and 100 μg/ml streptomycin and 2 mM l-glutamine (Gibco, Life Technologies). The cells were maintained in 75 cm^2^ flasks at 37°C in a humidified atmosphere containing 5% CO_2_ and split at 80% confluence every 5 days. Cells were seeded in 24-well plate (2 × 10^5^ cells) and incubated at 37°C overnight followed by cells pretreatment with complete DMEM containing high glucose concentration in order to downregulate autophagy (Kobayashi et al., 2012). After 6 h, cells were treated with different concentrations of postbiotics obtained from *Lactobacillus fermentum* BGHV110 strain (HV110) in order to select appropriate dose for further experiments. Postbiotic was dissolved in complete DMEM medium and added to the cells in specific final concentration. In all other experiments seeded cells were treated with 50 mM APAP (Sigma-Aldrich, Germany) alone or co-treated with 50 mM APAP and selected dose of lyophilized HV110. To analyze autophagic flux, simultaneously with treatments, cells were exposed to lysosomotropic agent chloroquine (Sigma-Aldrich) at a concentration of 25 μM, to inhibit autophagosome–lysosome fusion. After 16 h of incubation, cells were subjected to following analysis.

### Metabolic Activity of HepG2 Cells

Metabolic activity of HepG2 cells was examined by MTT [3-(4,5-dimethylthiazol-2-yl)-2,5-diphenyl tetrazolium bromide] assay (Serva, Electrophoresis GmbH, Heidelberg, Germany) as described by Mosmann (1983). After the treatment, the cells were washed with PBS and MTT dissolved in complete media was added at the final concentration of 0.5 mg/ml. After 4 h of incubation, at 37°C with 5% CO_2_, the media was aspirated and 10% SDS-0.01 N HCl was added to dissolve formazan. The absorbance was measured with a microplate reader (Tecan Austria GmbH, Grödig, Austria) at a wavelength of 570 nm. Results are presented as percentage of metabolic activity of treated cells compared to control.

### Cytotoxicity Assay

The level of cytotoxicity in the cell cultures was measured by lactate dehydrogenase (LDH) Cytotoxicity Assay Kit (Thermo Scientific) which detects LDH released from dead cells. After treatments, supernatants were collected and LDH activity was determined by following the manufacturer’s instructions. The absorbance was measured at 450 nm on a microplate reader (Tecan). Since there is an interference between APAP and LDH assay (Xu et al., 2003) only the treatments where APAP is used in the same dose are compared and results are presented as absorbance at 450 nm.

### Western Blotting

Following the different treatments, cells were lysed with RIPA buffer (50 mM Tris-HCl pH = 7.4; 150 mM NaCl; 1% NP-40; 0.25% sodium deoxycholate) containing Protease Inhibitor Cocktail Tablets (Roche, Basel, Switzerland) and 1 mM phenylmethyl sulfonyl fluoride (Sigma-Aldrich), for 30 min on ice. Cell lysate was centrifuged at 12000 rpm for 15 min at 4°C and the protein concentration was measured using Bradford reagent (Bio-Rad Laboratories). Total cell proteins (20 μg) were separated on 12% SDS-PAGE and transferred to 0.2 μm nitrocellulose membrane (GE Healthcare) using a Bio-Rad Mini *trans*-blot system (Bio-Rad, Hercules, CA, USA). In case of p62 detection, proteins were transferred to 0.45 μm PVDF membrane (Millipore Corporation, Billerica, MA, USA). Immunoblots were blocked in a 10% non-fat dry milk in TBS-Tween (50 mM Tris-HCl, pH 7.4; 150 mM NaCl, and 0.05% Tween-20) overnight at 4°C followed by 2 h incubation at room temperature with the primary antibodies; anti-LC3 (1:2000; Thermo Fischer Scientific), anti-p62 (1:1000; Progen Biotechnik GmbH, Heidelberg, Germany) and anti-β-actin (1:1000; Thermo Fischer Scientific). The membranes were subsequently washed and incubated with appropriate HPR-conjugated secondary antibodies (goat anti-rabbit; 1:10000; Thermo Fischer Scientific and goat anti-guinea pig; 1:10000; Novex Life Technologies) for 1 h at room temperature. Proteins were detected by enhanced chemiluminescence (Immobilon Western, Merck Milipore). The intensity of the bands was quantified using ImageJ software. p62 was normalized to β-actin loading control. Autophagy induction was measured by calculation of LC3-II/LC3-I ratio.

### Quantitative Real-time PCR

Total RNA was extracted from HepG2 cells as previously described by Lukic et al. (2013) with slight modifications. Cells were washed with PBS and lysed in denaturing solution (4 M guanidine thiocyanate, 25 mM sodium citrate, 0.1 M β-mercaptoethanol, 0.5% [wt/vol] *N*-lauroylsarcosinate sodium salt) followed by acid phenol (pH 4) extractions and isopropanol precipitation. cDNA was generated from 0.5 μg total RNA according to the reverse transcriptase manufacturer’s protocol (Thermo Scientific). Quantitative PCR was carried out on 7500 real-time PCR system (Applied Biosystems, Waltham, MA, USA) using KAPA SYBR Fast qPCR Kit (Kapa Biosystems, Wilmington, MA, USA) under the following conditions: 3 min at 95°C activation, 40 cycles of 15 s at 95°C and 60 s at 60°C. All used primers (**Table 1**) were purchased from Thermo Fisher Scientific.

**Table 1:** The list of primers used in this study.

Primer name	Primer sequence 5′–3′	Reference
β-Actin forward	TTGCTGACAGGATGCAGAAGGAGA	Li et al., 2015
β-Actin reverse	TCAGTAACAGTCCGCCTAGAAGCA	
BECN1 forward	CTGGGACAACAAGTTTGACCAT	Liu et al., 2014
BECN1 reverse	GCTCCTCAGAGTTAAACTGGGTT	
ATG5 forward	CACAAGCAACTCTGGATGGGATTG	He et al., 2013
ATG5 reverse	GCAGCCAC GGACGAAACAG	
p62/SQSTM forward	GCCAGAGGAACAGATGGAGT	Sahani et al., 2014
p62/SQSTM reverse	TCCGATTCTG GCATCTGTAG	
PINK1 forward	GGGGAGTATGGAGCAGTCAC	Kim et al., 2013
PINK1 reverse	CATCAGGGTAGTCGACCAGG	

### Statistical Analysis

All data are presented as mean values ± standard error of the mean (SEM). One-way ANOVA with the Tukey’s *post hoc* test were used to compare multiple groups. The differences between control and experimental groups were compared using Student’s *t*-test. Values at *p* < 0.05 or less were considered to be statistically significant. All experiments were repeated at least three times. Statistical analysis was carried out using SPSS 20.0 for Windows. Graphs were drawn in the GraphPad Prism software (trial version).

## Results

### HV110 Exhibits Dose-dependent Effect on Cell Viability

**FIGURE 1:**
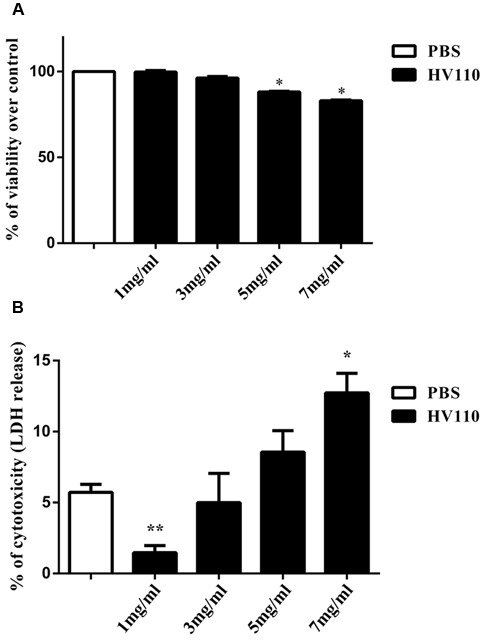
**Dose-dependent effect of HV110 on HepG2 cells viability after 16 h of treatment assessed by MTT assay** (**A**; *n* = 3) and LDH assay (**B**; *n* = 3). All values are presented as mean ± SEM. Student’s *t*-test was used to compare treated groups relative to control (*p < 0.05, **p < 0.01).

We initially investigated in which way HV110 affects metabolic activity of HepG2 cells. The results of MTT assay showed dose-dependent effect of HV110 on HepG2 metabolic activity. Doses of 5 and 7 mg/ml of HV110 significantly (*p* < 0.05) decreased cell metabolic activity to 88.11 ± 0.43% and 83.06 ± 0.35%, respectively (**Figure 1A**). As detected decrease in metabolic activity could point on cell death, the level of LDH released in the cell culture was examined in order to determine the HV110 cytotoxicity. Only when applied in dose of 7 mg/ml, significantly higher (*p* < 0.05) LDH level in supernatants of the treated cells was detected in comparison to control (**Figure 1B**). Since the dose of 3 mg/ml didn’t change cell metabolic activity or cause cell damage, this dose was used in further experiments.

### HV110 Protects Cells against APAP-Induced Hepatotoxicity

**FIGURE 2:**
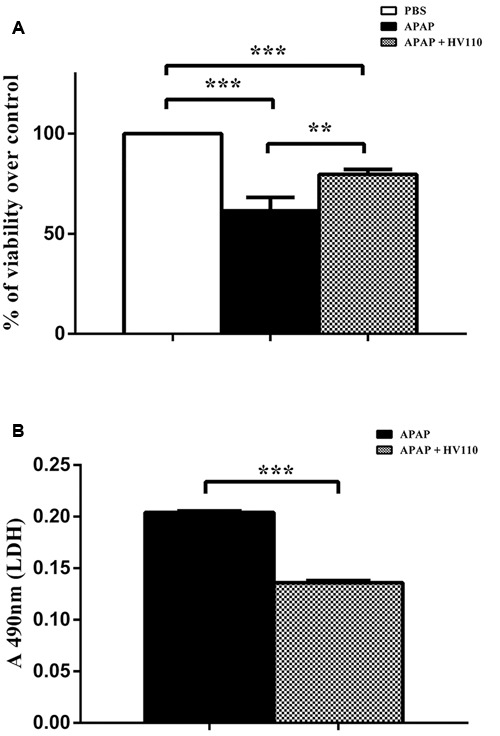
**HV110 protects cells against APAP-induced hepatotoxicity.** Viability of HepG2 cells treated with 50 mM APAP and 3 mg/ml of HV110 for 16 h was examined by MTT (**A**, *n* = 4) and LDH assay (**B**, *n* = 3). LDH results are presented only as absorbance values indicating that higher absorbance correlates with the elevated cytotoxicity. All values are mean ± SEM. One-way ANOVA with the Tukey’s *post hoc* test was used to compare multiple groups (**p < 0.01, ***p < 0.001).

In order to explore the potential cyto-protective role of selected dose of HV110 in APAP-induced hepatotoxicity, cell viability in the presence of APAP was assessed using MTT and LDH assays. As expected, MTT assay shows that APAP in a dose of 50 mM significantly (*p* < 0.001) reduced cell viability to 61.5 ± 6.65%. Interestingly, the significant (*p* < 0.01) increase in cell viability to 79.7 ± 2.47% was observed in the APAP/HV110 co-treated cells, compared to APAP treated cells (**Figure 2A**). In parallel, LDH release in the media was measured and the results showed significantly lower (*p* < 0.001) toxic effect of APAP in the presence of HV110. Due to interference between APAP and LDH assay, the results of LDH assay are presented only as absorbance values, indicating that higher absorbance correlate with the increased cytotoxicity (**Figure 2B**).

### The Influence of HV110 on Autophagy in HepG2 Cells

**FIGURE 3:**
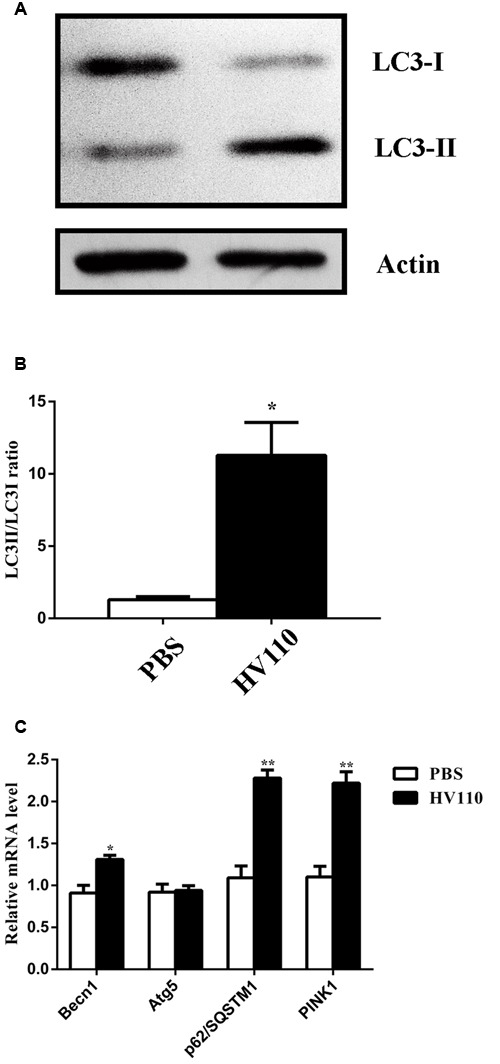
**The influence of HV110 on autophagy in HepG2 cells.** Representative western blot **(A)** and densitometric analysis (**B**, *n* = 4) of LC3 conversion in HepG2 cells. Quantification of *BECN1*, *Atg5*, *p62/SQSTM1* and *PINK1* mRNA levels (**C**, *n* = 3). HepG2 cells were treated with 3 mg/ml of HV110 for 16 h. Student’s *t*-test was used to compare experimental group relative to control (*p < 0.05, **p < 0.01).

One of the key cellular adaptive mechanisms involved in attenuation of APAP-induced liver injury is autophagy. To investigate whether autophagy is correlated with protective effect of HV110 on HepG2 cells several factors involved in autophagy process were monitored. At first, conversion of the soluble LC3-I to lipid-bound LC3-II form of LC3 protein, a commonly used marker of autophagosomes formation associated with number of autophagosomes, was assessed by Western blot analysis. The potential of HV110 alone to trigger protective autophagy in HepG2 cells was investigated. Results revealed the significant increase (*p* < 0.05) of LC3-II/LC3-I conversion compared to the control cells (**Figures 3A,B**). In addition, the expression of *BECN1* a gene involved in nucleation step, and *Atg5* gene, involved in elongation step of autophagy process, were determined. The results showed that the expression of *BECN1* was significantly increased (*p* < 0.05) in cells treated with HV110, while the expression of *Atg5* remained unchanged, in comparison to control untreated cells (**Figure 3C**).

**FIGURE 4:**
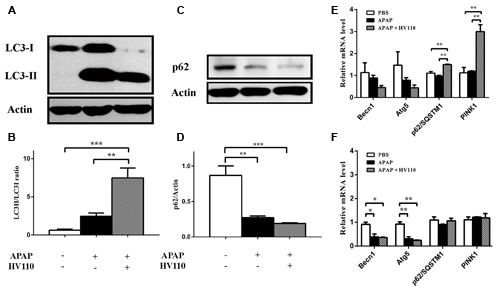
**Correlation between autophagy and HV110 protective effects.** Evaluation of LC3 conversion and p62 degradation by immunoblot **(A,C)** and densitometric analysis (**B**, *n* = 5; **D**, *n* = 4) in HepG2 cells treated with 50 mM APAP and 3 mg/ml of HV110 for 16 h. The mRNA expression levels of *BECN1*, *Atg5*, *p62/SQSTM1* and *PINK1* after 6 h (**E**, *n* = 3) and 16 h (**F**, *n* = 3) of treatments with APAP and HV110. Values are expressed as mean ± SEM. One-way ANOVA with the Tukey’s *post hoc* test were used to compare multiple groups (*p < 0.05, **p < 0.01, ***p < 0.001).

Further, the LC3-II/LC3-I ratio in APAP/HV110 co-treatment of HepG2 cells was followed. The results showed significant increase of LC3-II/LC3-I conversion in APAP/HV110 treated cells compared to cells treated with APAP alone (*p* < 0.01) and untreated control cells (*p* < 0.001), respectively (**Figures 4A,B**). Interestingly, although the treatment with APAP alone induced conversion of LC3 protein, it should be noted that this induction was not statistically significant in comparison to untreated cells.

Next, we investigated p62/SQSTM1 protein degradation by autophagy machinery. As a cargo receptor, p62/SQSTM1 binds to LC3 protein and contributes to clearance of ubiquitinated proteins. Consistent with the above mentioned results, APAP treatment as well as APAP/HV110 co-treatment caused significant degradation of p62/SQSTM1 protein compared to control, respectively (*p* < 0.01, *p* < 0.001). However, difference in p62/SQSTM1 degradation between APAP treatment and APAP/HV110 co-treatment is visible on western blot, but didn’t reach statistically significance (**Figures 4C,D**).

**FIGURE 5:**
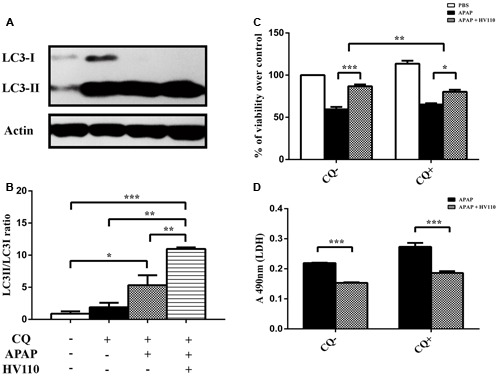
**Autophagy flux assessment.** Representative western blot **(A)** and densitometric analysis (**B**, *n* = 3) of LC3 conversion in HepG2 cells treated for 16 h with 50 mM APAP and 3 mg/ml of HV110 in the absence or presence of chloroquine (CQ, 25 μM). In parallel, cell viability of HepG2 cells were evaluated using MTT assay (**C**, *n* = 3) and LDH assay (**D**, *n* = 3). All values are presented as mean ± SEM. For comparison of multiple groups, one-way ANOVA with the Tukey’s *post hoc* test were used (*p < 0.05, **p < 0.01, ***p < 0.001).

Autophagosomes’ accumulation could be a consequence either of autophagy activation or inhibition of downstream autophagy steps. Therefore, assessment of autophagy flux, which reflects the dynamics of the process, is essential. Results of autophagy flux monitoring showed significant increase conversion of LC3 marker and accumulation of LC3-II in the APAP/HV110 co-treated cells compared to chloroquine treated control (*p* < 0.01; **Figures 5A,B**), supporting the above mentioned evidence of autophagy activation.

However, levels of mRNA of *BECN1* and *Atg5* genes were decreased after 6 h and reached statistical significant decrease after 16 h of treatment (*p* < 0.05, *p* < 0.01) with no differences between APAP and APAP/HV110 co-treated groups (**Figures 4E,F**).

### Autophagy Inhibition Decreases the Protective Effect of HV110

With the aim of the final confirmation of involvement of HV110-induced autophagy in protective effect on HepG2 cells against APAP, the autophagy was inhibited by chloroquine. The results of MTT assay showed that chloroquine added to the APAP/HV110 co-treated HepG2 cells decreased cell survival compared to the same treatment without chloroquine. More precisely, the difference between bars representing the percentage of viable cells after APAP/HV110 co-treatment and the percentage of viable cells after APAP treatment, without added chloroquine, was significantly higher (27.42 ± 1.86%) than in the presence of chloroquine (15.07 ± 1.21%) (*p* < 0.01; **Figure 5C**). This result supports our assumption of autophagy involvement in survival of cells treated with HV110. Additionally, LDH levels in supernatants of the cells treated with APAP, HV110 and chloroquine were much higher compared to treatment with no chloroquine added, but statistical significance wasn’t achieved (**Figure 5D**). Considering the fact, that APAP/HV110 co-treated cells in the presence of chloroquine, still exhibit significantly higher viability rate compared to only APAP treated cells (MTT; *p* < 0.05 and LDH; *p* < 0.001) in the presence of chloroquine, the additional mechanism(s) involved in protective effect of HV110 on APAP-induced hepatotoxicity could be assumed.

### Gene Expression Profile Revealed the Activation of PINK1 Autophagy Pathway

To analyze whether the PINK1-Parkin signaling pathway could be responsible for autophagy activation, we followed the expression of *PINK1* and *p62/SQSTM1* genes by qPCR. Results revealed that *PINK1* and *p62/SQSTM1* mRNAs were significantly induced in cells treated only with HV110 (*p* < 0.01) (**Figure 3C**).

Next, the expression profile of *PINK1* and *p62/SQSTM1* in APAP-induced hepatotoxicity was studied. The expression of the analyzed genes was not changed after the APAP treatment, regardless of the exposure time. Interestingly, the levels of *PINK1* and *p62/SQSTM1* mRNAs were increased in cells co-treated with both APAP and HV110 for 6 h, compared to control and APAP treated cells (*p* < 0.01; **Figure 4E**). After 16 h of APAP/HV110 treatment, the genes’ expression returned to the control level (**Figure 4F**).

## Discussion

The literature data regarding the involvement of probiotics in autophagy activation are still limited. Most of the research in this field has been focused on the influence of pathogenic bacteria on autophagy machinery, while little space was given to the research on potential of beneficial bacteria to stimulate autophagy (Escoll et al., 2016; Zhang et al., 2016). This is the first report indicating that postbiotic HV110, originated from *Lactobacillus fermentum* BGHV110 strain, is potent inducer of autophagy in HepG2 cells, as demonstrated by increased LC3 lipidation and mRNA expression of *BECN1*, *PINK1*, and *p62/SQSTM1*. Bacterial lysates are rich in different pathogen associated molecular patterns (PAMPs) which can trigger autophagy through Toll-like receptor (TLR) signaling (Delgado et al., 2008). TLRs play an important role in liver physiology and pathophysiology due to the liver’s exposure to gut-derived bacterial products and they are expressed in all cells present in the liver (Seki and Brenner, 2008; Mencin et al., 2009). Also, research over the last few years identified another class of pattern recognition receptors (PRRs), NOD-like receptors, involved in autophagy (Oh and Lee, 2014). On the other side, it has been described that overstimulation of PRRs can lead to induction of apoptosis. For example, TLR2 and TLR4 ligands present in the mycobacterial cell wall were identified as active ingredients of BCG treatment of superficial bladder tumors (Salaun et al., 2007; Subramaniam et al., 2016). This could be a reason for dose dependent decreased of cell viability caused by HV110 which was obtained in this study. However, involvement of PRRs in HV110 induced autophagy and impact on cell viability should be tested in further experiments.

Our finding that HV110 exhibits potential to induce protective autophagy served as the starting point to examine its cyto-protective effects against APAP-induced hepatotoxicity, which was shown that could be alleviated by autophagy induction (Ni et al., 2012). APAP exerts its toxic effects by two mechanisms: by CYP450-dependent NAPQI generation and by formation of PAP, as the result of APAP deacetylation (Miyakawa et al., 2015). CYP450-dependent pathway is activated immediately after APAP exposure and it lasts for the first several hours of exposure. However, after prolonged exposure to APAP, PAP-mediated pathway is activated with higher impact on cell viability compared to CYP450-dependent pathway. We thus assume that, in spite of lower expression of CYP450 enzymes in HepG2 cells (Westerink and Schoonen, 2007), our experimental setup provided enough and sustainable damage in HepG2 cells.

Though numerous studies investigated the effects of cytoprotective agents applied to hepatocytes before or after the addition of cytotoxic agents, our study was concerned with simultaneous HV110/APAP application. According to the results presented by Sharma et al. (2011) post-treatment with cytoprotective probiotics could not effectively reduce hepatocyte damage after APAP exposure, though pre and co-treatment were shown to be effective in the same study. This indicates that cell damage inflicted by high APAP doses is irreversible, as also evident from case studies reporting liver failure after intake of high APAP doses. Eventually, incorporation of postbiotics in formulations containing hepatotoxic drugs could significantly reduce the side effects and the degree of intoxication.

HV110 succeeded to alleviate APAP induced cell damage, as evidenced from MTT and LDH assays. Although APAP exposure *per se* induced protective autophagy in HepG2 cell line, induction of autophagy was threefold higher in APAP/HV110 co-treated cells, according to the degree of LC3 protein conversion and 1.5-fold higher based on the p62/SQSTM1 protein degradation. Chloroquine also favored the formation of autophagic vesicles containing cellular components in cells stimulated with HV110. Moreover, presence of chloroquine resulted in decreased survival of the cells exposed to APAP/HV110, suggesting the role of HV110-induced autophagy in the cells’ protection. However, after chloroquine treatment, percentage of viable cells exposed to APAP/HV110 has not decreased to the viability level of those treated only with APAP, suggesting involvement of other protective mechanisms.

According to the results of mRNA expression, APAP/HV110 treated cells elevated mRNA levels of *p62/SQSTM1* and *PINK1* after 6 h. The main role of PINK1 in cells is to promote Parkin-mediated mitophagy by recruiting Parkin to damaged mitochondria (Williams and Ding, 2015). Along with its protective function and the potential to activate mitophagy, it was shown that PINK1 may also have an important role in activation of basal and starvation-induced autophagy by interacting with Beclin-1 protein (Michiorri et al., 2010). On the other hand, p62/SQSTM1 is a molecular adapter between degradation substrates and molecules involved in autophagosome formation and antioxidant defense (Lamark et al., 2009; Rautou et al., 2010; Ichimura et al., 2013). Elevated *p62/SQSTM1* mRNA synthesis in cells co-treated with APAP/HV110 implies activation of NF-E2-related factor 2 (Nrf-2), an important transcription factor that regulates the expression of antioxidant specific genes. Recent data showed that p62/SQSTM1 also plays important role in regulation of Nrf2 activity. The p62 binds to Kelch-like ECH-associating protein 1 (Keap1) causing release of Nrf2 and consequent transcription of genes, including those involved in xenobiotic and ROS (reactive oxygen species) detoxification (Ichimura et al., 2013). Jones et al. (2015) provided evidence that lactobacilli can elicit their beneficial influences in the gut through direct contact with NADPH oxidases on a cell surface, resulting in ROS generation and consequential activation of Nrf2 signaling. However, according to our knowledge this is the first study that reports link between postbiotics and p62/SQSTM1 as possible new mechanism of postbiotics activation of Nrf2 pathway in hepatocytes. *PINK1* gene upregulation suggests that HV110 treatment triggers a low sub-lethal level of oxidative stress that could be reflected at the level of transcription of *PINK1* which is highly sensitive to subtle changes in intracellular environment.

Although APAP/HV110 co-application induced changes in *PINK1* and *p62/SQSTM1* mRNA expression after 6 h treatment, the values returned to basal levels after prolonged treatment (16 h). It was shown that antioxidants produced by cells as response to oxidative damage, in this case caused by APAP, inhibit Nrf2 dependent gene transcription, via negative feedback (Murata et al., 2015). Considering the fact that *PINK1* regulation, as well as regulation of *p62/SQSTM1* transcription is dependent on Nrf2 protein, this could explain downregualtion of *PINK1* and *p62/SQSTM* mRNA expression after 16 h treatment, which was obtained in this study (Jain et al., 2010; Murata et al., 2015). Interestingly, we did not detect any changes in *PINK1* and *p62/SQSTM1* expression, in cells treated only with APAP, neither after 6 h nor after 16 h of treatment. This suggests that mechanisms which lead to acute cellular response were more intensively activated in the presence of HV110. It could be assumed that rapid activation of these mechanisms might have elevated cellular defensive system, including autophagy response which aided cellular survival, as demonstrated in MTT and LDH assays. All above mentioned results are consistent with the concept of “hormesis,” a response to xenobiotic and environmental stimuli, where low stress levels are protective against more destructive stimuli. This concept has generally been given as an explanation for beneficial effects of microbes upon the host (Jones et al., 2015).

In spite of their well-known role in autophagy induction, we surprisingly noticed a decrease of *BECN1* and *Atg5* transcription in APAP treated cells, for which increased LC3 lipidation was obtained. This was irrespective of HV110 presence. Beclin-1, in complex with class III phosphatidylinositol 3-kinase (PI3K) is crucial for the nucleation phase of autophagosome formation, while Atg5 conjugates to Atg12 and facilitates the lipidation of LC3 protein (Rautou et al., 2010; Otomo et al., 2013). Aside from being indispensable for autophagy, Beclin-1 has additional roles not related to autophagosomes formation. Beclin-1 is known as haplo-insufficient tumor-suppressor and accumulating evidence of data suggests its down-regulation in cancers (Li et al., 2013; Huang et al., 2014). Findings of Li et al. (2013) show that down-regulation of Beclin-1 triggered autophagy, decreased apoptosis and stimulated proliferation of cells exposed to gemcitabine in Miapaca2 tumor cells. Therefore, lower levels of *BECN1* mRNA in APAP-treated cells could be explained as a cellular attempt to overcome toxicity caused by very high APAP dose. In the case of *Atg5* gene downregulation, the results could be explained by the role of Atg5 protein to regulate its own transcription through a feedback inhibition loop, as demonstrated by Hu et al. (2011).

## Conclusion

Our study demonstrated protective effect of autophagy activated by postbiotic HV110 originated from *Lactobacillus fermentum* BGHV110 strain in APAP induced cytotoxicity in HepG2 cells. To the best of our knowledge, this is the first study to correlate autophagy inductive potential of lactobacilli to their protective effects against drug-induced toxicity. Taken together, this could be of special relevance for designing of new analgetic drug formulations with added postbiotic for prevention of possible hepatotoxic side effects, although the safety and health promoting efficacy of such drugs should be further tested.

## Author Contributions

MD: performed main work, analyzed, interpreted the data and draft the work; JL: conception and design of experiments, performed part of the experiments, analyzed, interpreted the data and critically revised the manuscript; JD: performed part of the experiments, analyzed and interpreted the data; MM: supervised the work, analyzed the data and critically revised the manuscript; IS: analyzed, interpreted and critically revised the manuscript; NG: supervised the work, analyzed and interpreted the data, draft the work; JB: conception and design of the work, supervised the work, analyzed and interpreted the data and critically revised the manuscript. All authors finally approved the version to be published and agreed to be accountable for all aspects of the work in ensuring that questions related to the accuracy or integrity of any part of the work are appropriately investigated and resolved.

## Conflict of Interest Statement

The authors declare that the research was conducted in the absence of any commercial or financial relationships that could be construed as a potential conflict of interest.

## Footnotes

1 http://www.ncbi.nlm.nih.gov/BLAST
